# Comprehensive identification of alternative back-splicing in human tissue transcriptomes

**DOI:** 10.1093/nar/gkaa005

**Published:** 2020-01-24

**Authors:** Peng Zhang, Xiao-Ou Zhang, Tingting Jiang, Lingling Cai, Xiao Huang, Qi Liu, Dan Li, Aiping Lu, Yan Liu, Wen Xue, Peng Zhang, Zhiping Weng

**Affiliations:** 1 Department of Thoracic Surgery, Clinical Translational Research Center, Shanghai Pulmonary Hospital, School of Life Sciences and Technology, Tongji University, Shanghai 200092, China; 2 Program in Bioinformatics and Integrative Biology, University of Massachusetts Medical School, Worcester, MA 01605, USA; 3 RNA Therapeutics Institute, University of Massachusetts Medical School, Worcester, MA 01605, USA

## Abstract

Circular RNAs (circRNAs) are covalently closed RNAs derived from back-splicing of genes across eukaryotes. Through alternative back-splicing (ABS), a single gene produces multiple circRNAs sharing the same back-splice site. Although many ABS events have recently been discovered, to what extent ABS involves in circRNA biogenesis and how it is regulated in different human tissues still remain elusive. Here, we reported an in-depth analysis of ABS events in 90 human tissue transcriptomes. We observed that ABS occurred for about 84% circRNAs. Interestingly, alternative 5′ back-splicing occurs more prevalently than alternative 3′ back-splicing, and both of them are tissue-specific, especially enriched in brain tissues. In addition, the patterns of ABS events in different brain regions are similar to each other and are more complex than the patterns in non-brain tissues. Finally, the intron length and abundance of *Alu* elements positively correlated with ABS event complexity, and the predominant circRNAs had longer flanking introns and more *Alu* elements than other circRNAs in the same ABS event. Together, our results represent a resource for circRNA research—we expanded the repertoire of ABS events of circRNAs in human tissue transcriptomes and provided insights into the complexity of circRNA biogenesis, expression, and regulation.

## INTRODUCTION

Circular RNAs (circRNAs) are a class of abundant, endogenous, non-polyadenylated RNAs with a covalently closed, continuous loop structure (1). Discovered over 20 years ago, they have become widely detected across the eukaryotic tree of life only in recent years due to advances in high-throughput sequencing technologies (2). Unlike mRNAs and lncRNAs, which undergo canonical splicing, circRNAs are produced from pre-mRNAs and pre-lncRNAs by back-splicing, whereby a downstream 5′ splice (donor) site is joined with an upstream 3′ splice (acceptor) site (3). Factors that bring the 5′ and 3′ back-splice sites into close proximity promote circRNA production, and such factors include inverted *Alu* repeats (IR*Alu*) in the flanking introns or dimerization of RNA-binding proteins bound to these introns (4). Increasing evidence indicates that circRNAs are regulated across tissue types, developmental stages, and disease conditions and they play important roles in critical biological processes including transcription, mRNA splicing, RNA decay, and translation (5–7) (for reviews, see (8–10)).

**Figure 1.**
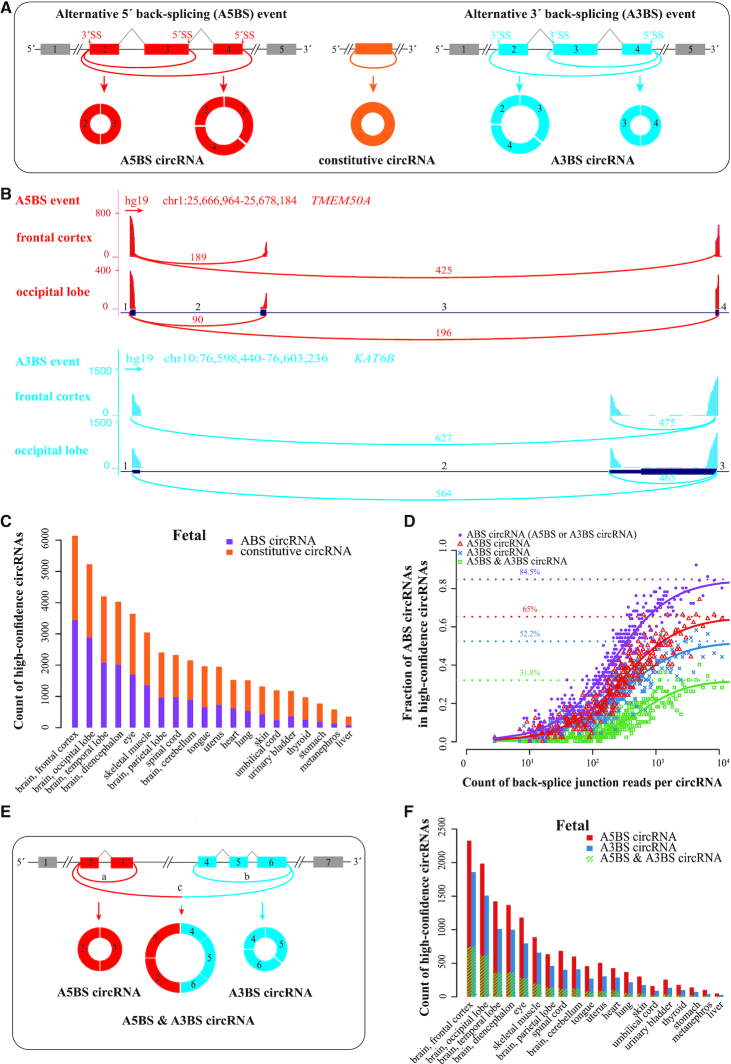
ABS events of circRNAs are prevalent in human tissue transcriptomes. (**A**) Schematic diagrams of two types of ABS events and three types of circRNAs. Colored boxes, exons. Black lines, introns. Gray polylines, canonical splicing. Colored arcs, back-splicing. Circles, circRNAs. (**B**) An example A5BS event (top panel) and an example A3BS event (bottom panel) in genes *TMEM50A* and *KAT6B* in human frontal cortex and occipital lobe tissues, respectively. The number of back-splice junction reads for each circRNA is displayed as red text above the corresponding arc. The indices of circRNA-forming introns are labeled in black text above the introns: introns 1, 2, 3 and 4 for *TMEM50A* and introns 1, 2 and 3 for *KAT6B*. Red and blue arcs, back-splicing. (**C**) The count of ABS and constitutive circRNAs among high-confidence circRNAs (≥ 0.1 RPM) for human fetal tissues. (**D**) Mean fraction of ABS circRNAs in groups of 50 circRNAs, binned by their pooled back-splice junction reads. The fraction of ABS circRNAs at sufficient sequencing depth was estimated to be the asymptote of the best-fit sigmoid curve (purple for ABS circRNAs, red for A5BS circRNAs, blue for A3BS circRNAs, and green for A5BS & A3BS circRNAs). Points show the fraction of ABS circRNAs in each bin. (**E**) Schematic diagrams of an example A5BS & A3BS circRNA. Colored boxes, exons. Black lines, introns. Colored arcs, back-splicing. Circles, circRNAs. (**F**) The count of A5BS & A3BS circRNAs as special cases of A5BS circRNAs and A3BS circRNAs for human fetal tissues.

A single gene locus can produce multiple circRNAs sharing the same back-splice site through a mechanism called alternative back-splicing (ABS), due to the competition among inverted complementary sequences across introns that bracket the circRNA-forming exons (11). There are two types of ABS events (Figure 1A), alternative 5′ back-splicing (A5BS) and alternative 3′ back-splicing (A3BS). In an A5BS event, two or more downstream 5′ back-splice sites are alternatively joined with the same upstream 3′ back-splice site, while for A3BS circRNAs, the same downstream 5′ back-splice site is alternatively joined with two or more upstream 3′ back-splice sites. We define constitutive circRNAs as those that do not share a back-splice site with other circRNAs. An earlier study expanded the complexity of circRNA formation and concluded that ABS events were highly diverse among various human cell lines (12). However, how prevalent ABS events are and how they are regulated in human tissues remain to be explored.

Here, we comprehensively analyzed 90 RNA-seq datasets in 37 tissues from 15 human donors (20 fetal from 11 donors and 18 adult tissues each from four donors) generated by the ENCODE project and built an atlas of ABS events across various human tissue transcriptomes. We found that ABS events were prevalent and most ABS events were tissue-specific—correlations among ABS profiles in the same tissue from different individuals were higher than correlations among different tissues from the same individual. We further investigated the sequence features correlated with ABS events and the choice of a back-splice site. Together, the landscape of ABS events in human tissue transcriptomes provides a valuable resource for depicting the complexity of circRNA biogenesis, expression, and regulation.

## MATERIALS AND METHODS

### Identifying ABS events and ABS circRNAs

We used the CIRCexplorer2 pipeline (12) to identify circRNAs in 37 types of human tissues using ribo–, total-RNA-seq datasets generated by the ENCODE Consortium (Supplementary Table S1). We instructed CIRCexplorer2 to use a two-step mapping strategy to identify high-confidence back-splice junction reads. First, RNA-seq reads from each sample were mapped to the GRCh37/hg19 human reference genome with the UCSC gene annotation (knowGene.txt updated at 17 July 2015) by TopHat2 (13) (Version 2.1.0; parameters: -a 6 -g 1 --microexon-search -m 2). Second, the unmapped reads were then extracted and mapped to the same human reference genome by TopHat-Fusion (14) (Version 2.1.0; parameters: --fusion-search --keep-fasta-order --bowtie1 --no-coverage-search). Back-splice junction reads were then identified and compared against the same UCSC gene annotation to determine the genomic coordinates of the 5′ and 3′ back-splice sites for each circRNA using CIRCexplorer2. To quantify the expression levels of circRNAs, their numbers of back-splice junction reads were normalized to Reads Per Million mapped reads (RPM). For datasets with biological replicates, RPMs were averaged across replicates for each circRNA. The circRNAs with ≥ 0.1 RPM were defined as high-confidence circRNAs. An ABS event is defined as a group of circRNAs that share the same 3′ back-splice site (A5BS event) or 5′ back-splice site (A3BS event) according to the genomic coordinates of the circRNAs. A high-confidence ABS event was defined as containing two or more circRNAs that were ≥ 0.1 RPM in at least one tissue. High-confidence circRNAs were defined as ABS circRNAs if they were involved in at least one high-confidence ABS event, otherwise as constitutive circRNAs. Most of our analyses were focused on high-confidence ABS events and high-confidence circRNAs.

### Estimating the fraction of ABS circRNAs

The back-splice junction reads (in RPM) were pooled together for each high-confidence circRNA to obtain an estimate of its total abundance across all human tissues, and high-confidence circRNAs were sorted by this total abundance and then binned. The fraction of ABS circRNAs was calculated for each bin. The fraction of ABS circRNAs in a bin positively correlated with the normalized back-splice junction-read coverage in the bin (Supplementary Figure S1B), so there must be ABS circRNAs not yet identified because of insufficient sequencing depth. To estimate the fraction of ABS circRNAs that would be observed given saturating coverage, a sigmoid curve of the form $F( x ) = \frac{\alpha }{{1 + {e^{ - ( {x - m} ) \times n}}}}$was fitted to the fraction of ABS circRNAs ($F(x)$) in each bin as a function of the back-splice junction-read coverage ($x$) with two constants $m$ and $n$, and the upper asymptote (${\rm{\alpha }}$) represented the fraction of ABS circRNAs at saturating coverage. Similar estimates of ${\rm{\alpha }}$ were obtained at different bin sizes (50–500 circRNAs per bin), suggesting the results were robust to the choice of bin size (Supplementary Figure S1D).

### Identifying tissue-specific ABS events

**Figure 2.**
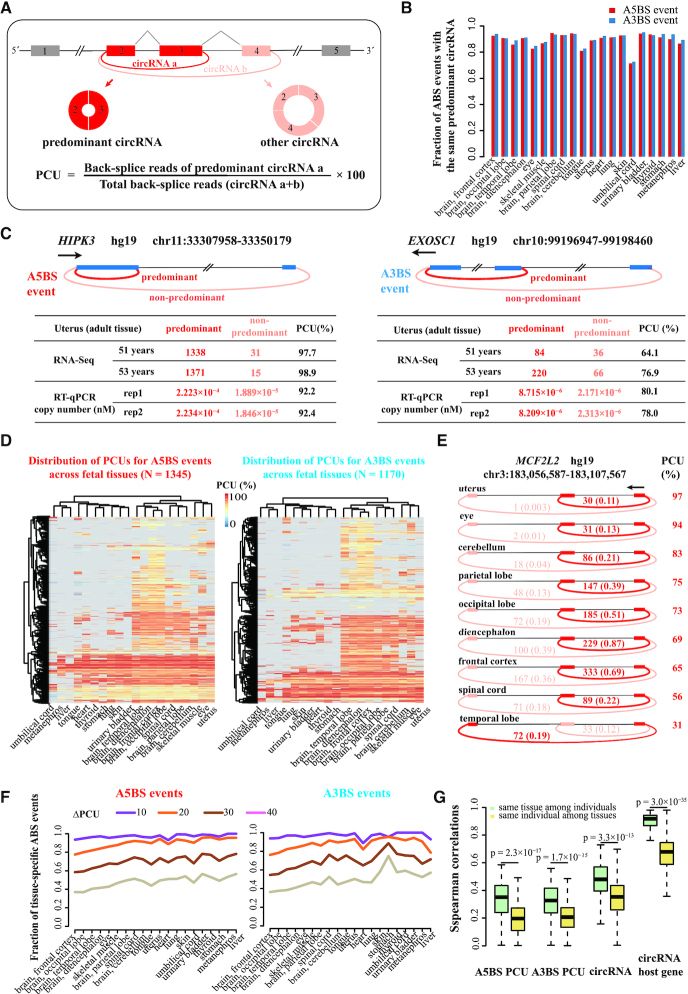
The extent of tissue-specific and individual-specific ABS events in human fetal and adult tissue transcriptomes. (**A**) A schematic diagram of the PCU metric for the predominant circRNA. Colored boxes, exons. Black lines, introns. Gray polylines, canonical splicing. Colored arcs, back-splicing. Circles, circRNAs. (**B**) The fraction of ABS events in fetal tissues with the same predominant circRNA for A5BS events and A3BS events separately. (**C**) The PCUs in adult human uterus for an A5BS event in the *HIPK3* gene (left panel) and an A3BS event in the *EXOSC1* gene (right panel) calculated using RNA-seq data in two tissue samples and measured using RT-qPCR in one tissue sample, respectively. (**D**) The distribution of PCUs for A5BS events (left panel) and A3BS events (right panel) across human fetal tissues. (**E**) Visualization of A5BS events produced from the *MCF2L2* gene across human fetal tissues. The A5BS events were sufficiently abundant for their PCUs to be calculated in 9 of 20 fetal tissues. The two A5BS circRNAs are indicated by dark red and light red arcs with raw back-splice junction reads (average RPM of two biological replicates) above the arcs. The PCU of the ABS event in each tissue is provided to the right. Colored arcs, back-splicing. (**F**) The fraction of tissue-specific A5BS events (left panel) and A3BS events (right panel) among human fetal tissues with ΔPCU ranging from 10 to 40. (**G**) Spearman correlations of PCUs for ABS events, circRNAs, and their host genes in the same tissue among different individuals and in the same individual among different tissues, respectively.

To systematically evaluate each ABS event across human tissues, we defined the Percent Circularized-site Usage (PCU) of the predominant circRNA of an ABS event as the ratio of the number of back-splice junction reads of the predominant circRNA to the total number of back-splice junction reads of all ABS circRNAs that share the same back-splice site (Figure 2A illustrates the situation using an ABS event with just two ABS circRNAs). The predominant circRNA was defined as the most abundant circRNA for the majority of tissues in a specific ABS event (Supplementary Figure S4A). The definition was performed for fetal tissues and adult tissues separately, and for the four adult individuals separately as well. We computed the PCUs for high-confidence ABS events in each tissue. To ensure the reliability of the PCUs for ABS events, we only calculated PCUs for the ABS events which contained at least one high-confidence circRNA (≥ 0.1 RPM) in the tissue (Supplementary Figure S4A). ABS events that exhibited a ΔPCU higher than a preset cutoff between at least one pair of tissues were considered to be tissue specific.

### Identifying ABS events in brain and non-brain tissues

The brain tissues in our study referred to seven human fetal tissues including frontal cortex, occipital lobe, temporal lobe, diencephalon, parietal lobe, spinal cord and cerebellum. Meanwhile, non-brain tissues included 13 human fetal tissues: eye, skeletal muscle, tongue, uterus, heart, lung, skin, umbilical cord, urinary bladder, thyroid, stomach, metanephros, and liver. The ABS events in brain tissues refer to those that contained two or more circRNAs that were ≥0.1 RPM in at least one brain tissue. Similarly, the ABS events in non-brain tissues contain two or more high-confidence circRNAs in at least one non-brain tissue.

### Quantifying the expression levels of circRNAs’ host genes

To quantify the expression level of a circRNA’s host gene, we used Cufflinks (15) (Version 2.2.1; default parameters) to calculate fragments per kilobase of transcript per million mapped fragments (FPKM) for each gene in each tissue using the same human UCSC gene annotation described above.

### Genomic features of a circRNA’s flanking introns

Sequences of the introns flanking a circRNA were extracted from the same human UCSC gene annotation described above. Control introns were randomly selected from the remaining introns in the circRNA’s host gene. The lengths of upstream and downstream flanking introns of three ABS event complexity classes and control introns were calculated separately.

### Quantifying *Alu* elements in a circRNA’s flanking introns

The annotation of human repetitive elements (rmsk.txt, updated on 16 July 2014) was used to determine the distribution of *Alu* elements in circRNA-flanking introns. Bedtools intersect (16) (v2.25.0; default parameters) was used to identify the *Alu* elements overlapping with circRNA-flanking introns. The count of *Alu* elements in upstream and downstream flanking introns of three ABS event complexity classes and control introns were calculated separately.

### Quantifying CSI of orientation-opposite complementary sequences across ABS circRNAs

We used the Complementary Sequence Index (CSI) to quantify RNA pairing capacity of orientation-opposite complementary sequences across predominant and other circRNAs separately (17). All factors that may contribute to RNA pairing formation, including sequence pairing strengths (BLAST Score), distances (Symmetry Length), and competition with other complementary sequences were considered in the evaluation. We used the maximum CSI to represent the strongest RNA pairing potential for each given flanking intron set.

### RT-qPCR

To make the real-time standard curve for quantifying the absolute copy numbers of ABS circRNAs, 1 μl of cDNA mixture was used to amplify each ABS circRNA with specific primers (Supplementary Table S4) using Phusion Flash High-Fidelity PCR Master Mix (Thermo Fisher). PCR reactions were carried out as follows: 98°C for 10 s, then 40 cycles of [98°C for 1 s, 55°C for 5 s and 72°C for 10 s], followed by a final 72°C extension for 3 min. The PCR products were purified by 1% agarose gel using a QIAquick Gel Extraction Kit (Qiagen), and the DNA concentration was measured by Nanodrop (Thermo Fisher). The DNA was diluted into 500×, 5000×, 50 000× and 500 000×. Real-time quantitative PCR analyses were performed with 1 ul of cDNA mixture or 1 ul purified DNA using SsoFast EvaGreen Supermix (Bio-Rad, Hercules, CA, USA) according to the manufacturer's protocol.

## RESULTS

### ABS events are prevalent

We used CIRCexplorer2 (12) to analyze 90 ribo–, total-RNA-seq datasets in 37 human tissues, including 20 fetal tissues, from 11 donors and 18 adult tissues, from four donors. These datasets were generated by the ENCODE Consortium and sequenced to great depths: 373M reads on average for the 20 fetal datasets and 108M reads on average for the 70 adult datasets (Materials and Methods; Supplementary Table S1). In total, we identified 201 490 circRNAs. To quantify the expression levels of these circRNAs, we normalized the number of back-splice junction reads by the sequencing depth of each dataset, expressed as RPM (Reads Per Million mapped reads), and defined circRNAs with ≥0.1 RPM as high-confidence circRNAs. Consistent with previous reports (18,19), we found that most of the circRNAs were expressed at low levels with only 8.7% of the circRNAs belonging to high-confidence circRNAs (Supplementary Table S2).

Although CIRCexplorer2 was shown to be one of the most sensitive and accurate circRNA identification algorithms (20–22), we tested another widely-used algorithm CIRI2 (23) to assess whether our results were affected by the choice of different methods. Among the 17 658 high-confidence circRNAs we defined using CIRCexplorer2, 16 061 (91%) of them could be detected by CIRI2, and the counts of back-splice junction reads detected using CIRCexplorer2 were highly correlated (Pearson correlation *r* = 0.99; *P*-value < 2.2 × 10^−16^) with the read counts identified using CIRI2 for each circRNA, indicating that the identification of high-confidence circRNAs using CIRCexplorer2 is robust.

It was reported earlier that trans-splicing events could confound circRNA identification (24). To examine this possibility for our analysis pipeline, we downloaded four RNA-seq datasets from Hs68 fibroblasts (19): two RNase R treated samples (SRA accessions SRR444974 and SRR445016; biological replicates of each other) and two untreated samples (SRR444655 and SRR444975; biological replicates of each other). We applied our pipeline to these datasets and computed the fold enrichment upon RNase R treatment for each identified circRNA. Among the 11 669 circRNAs we identified using untreated Hs68 fibroblasts, 191 were deemed high-confidence circRNAs (RPM ≥ 0.1) and all of them could also be detected and were highly enriched in the treated Hs68 fibroblasts (median fold enrichment = 11.9), suggesting that our pipeline was highly specific in identifying circRNAs even using samples not treated by RNase R. Furthermore, among the high-confidence circRNAs identified in our samples, 4788 circRNAs could also be identified in untreated Hs68 fibroblasts, and 4546 (94.9%) of them were significantly enriched in RNase R treated Hs68 fibroblasts (median fold enrichment = 12.3), suggesting that our circRNA identification pipeline is robust against trans-splicing events.

We then searched for 5′ or 3′ back-splice sites shared by multiple circRNAs to identified all ABS events in these 90 datasets (Materials and Methods). To prevent false positives, we only retained ABS events with two or more circRNAs that were classified as high-confidence in at least one tissue. Figure 1B shows an A5BS event in the gene coding Transmembrane Protein 50A (*TMEM50A*) and an A3BS event in Lysine Acetyltransferase 6B (*KAT6B*). For *TMEM50A*, the 5′ back-splice sites of intron 3 and intron 4 alternatively link to the upstream 3′ back-splice site of intron 1, while for *KAT6B*, two circRNAs use the same 5′ back-splice site of intron 3 to connect to the 3′ back-splice sites of intron 1 and intron 2, respectively. Both ABS events were supported by tens to hundreds of reads across several biosamples.

To quantify how prevalent ABS events are in circRNA biogenesis, we calculated the fraction of high-confidence circRNAs with ABS events in human fetal (Figure 1C) and adult tissues (Supplementary Figure S1A). The tissues with more high-confidence circRNAs had higher fractions of ABS circRNAs, and the fraction of ABS circRNAs correlated with the total back-splice junction reads of high-confidence circRNAs in the tissue (Pearson correlation coefficient *r* = 0.87, *P*-value < 2.2 × 10^−16^; Supplementary Figure S1B, C). Therefore, the ABS events for some of the low-abundance circRNAs have not yet been identified. To estimate the fraction of ABS circRNAs at a sufficient sequencing depth, we sorted and binned circRNAs by their back-splice junction reads and fitted the data to a sigmoid curve (Materials and Methods). The asymptotic fraction of ABS circRNAs reached 84.5%, and the fractions of A5BS circRNAs and A3BS circRNAs were 65% and 52.2%, respectively (Figure 1D; Supplementary Figure S1D). These results indicate that ABS events are prevalent in human circRNAs.

### Some circRNAs participate in both A5BS and A3BS events

We noticed that many circRNAs participated in both A5BS and A3BS events. As shown in the schematic diagram (Figure 1E), circRNAs a and c formed an A5BS event while circRNAs b and c formed an A3BS event, so circRNA c is an A5BS circRNA as well as an A3BS circRNA, and we defined circRNA c as an A5BS & A3BS circRNA. We quantified the factions of A5BS & A3BS circRNAs among ABS circRNAs in human fetal (Figure 1F) and adult tissues (Supplementary Figure S2A). The fraction of A5BS & A3BS circRNAs among high-confidence circRNAs positively correlated with the total number of high-confidence circRNA reads in a sample (Pearson correlation coefficient *r* = 0.93, *P*-value < 2.2 × 10^−16^; Supplementary Figure S2B), so we binned circRNAs by expression level and fitted the data to a sigmoid curve to determine the asymptotic fraction of A5BS & A3BS circRNAs at sufficient sequencing depth (Figure 1D). We estimated that 31.8% of high-confidence circRNAs were A5BS & A3BS circRNAs, suggesting that A5BS events and A3BS events often shared the same circRNAs.

### A5BS events are more prevalent than A3BS events

Intriguingly, A5BS circRNAs were more prevalent than A3BS circRNAs in all the human tissues that we examined (Wilcoxon signed-rank test *P*-value = 4.8 × 10^−5^; Figure 1F; Supplementary Figure S2A). The same trend was observed when A5BS and A3BS events were counted (Supplementary Figure S2C, D; Wilcoxon signed-rank test *P*-value = 9.4 × 10^−16^). In contrast, among alternative splicing events, those that involved alternative 3′ splice sites occurred more frequently than those that involved alternative 5′ splice sites (25). Considering that the back-splicing is also carried out by the spliceosomal machinery (26), we propose that the 5′-to-3′ transcriptional direction causes the higher frequency of A5BS than A3BS. A schematic figure illustrates that in an A5BS event, the shorter circRNA can form but the longer circRNA cannot while Pol II travels between the two alternative 5′ back-splice sites (Supplementary Figure S2E, left panel), but Pol II’s position does not favor one circRNA over the other circRNAs in the same A3BS events (right panel).

### Tissue-specific and individual-specific ABS events

Following previous work (12), we used PCU of the predominant circRNA to quantitatively compare ABS events across tissues (Figure 2A). The predominant circRNA was defined as the most abundant circRNA for the majority of tissues in one specific ABS event (Figure 2A). The definition was performed for fetal tissues and adult tissues separately. Defined as such, the predominant circRNA had the maximal abundance for the vast majority of ABS events in individual tissues (Figure 2B shows fetal tissues).

We evaluated the reproducibility and accuracy of our PCU method in three ways. First, we observed high correlations between the biological replicates of our samples (Spearman correlation ρ = 0.65 ± 0.06 for A5BS events and ρ = 0.62 ± 0.10 for A3BS events). Second, we applied the method to the aforementioned four Hs68 fibroblast samples, two with RNase R treatment and two without (19). The PCU values are highly correlated between the RNase R-treated and untreated samples (Spearman correlation ρ = 0.8 for A5BS events and ρ = 0.73 for A3BS events). Third, we randomly selected two ABS events in adult uterus—one A5BS event in the *HIPK3* gene and one A3BS event in the *EXOSC1* gene—for experimental validation. Each ABS event has two circRNAs—the predominant circRNA with a high RNA-seq read count and the other, non-predominant circRNA with a low RNA-seq read count (Figure 2C and Supplementary Figure S3A). We performed real-time quantitative PCR (RT-qPCR) experiments using primers specifically designed for each circRNA and then determined the copy number of the circRNA (Supplementary Figure S3B). The PCU values of these two ABS events determined using RT-qPCR agreed well with the corresponding PCU values computed using RNA-seq data (Figure 2C). These computational and experimental analyses indicate that our method can reproducibly and accurately identify the predominant circRNAs of ABS events and estimate their PCU values.

To explore the extent of tissue-specific ABS events, we defined a comprehensive set of 1345 A5BS events and 1170 A3BS events with sufficiently abundant circRNAs in fetal tissues and, likewise, 1026 A5BS events and 915 A3BS events in adult tissues (Materials and Methods; Supplementary Figure S4A). The distributions of PCUs across fetal tissues are shown as heatmaps for A5BS events (Figure 2D left panel) and A3BS events (Figure 2D right panel), indicating some ABS events had highly variable PCUs across different tissues. The distributions of PCUs across adult tissues further confirmed this phenomenon (Supplementary Figure S4B). For instance, The PCUs of an A5BS event in the *MCF2L2* gene varied from 97% in the uterus to 31% in the temporal lobe of the brain (Figure 2E). We further determined the fractions of tissue-specific ABS events, defined as those that differed in PCU between any pair of tissues (ΔPCU) by more than a certain cutoff, with the cutoff ranging from 10% to 40%. Although the fraction of tissue-specific ABS events decreased with stricter cutoffs, we still observed 36–75% ABS events being tissue-specific at ΔPCU ≥ 40 (Figure 2F).

Due to the scarcity of fetal tissue, some of the fetal samples were pooled from multiple individuals (Supplementary Table S3); thus, the PCU variations among fetal samples were confounded with the differences between tissues and between individuals. On the other hand, the 17 adult tissues were each from four individuals, providing a unique opportunity to assess the extent of PCU variations between tissues vs. between individuals. Thus, we computed the Spearman correlation coefficients among the two vectors of PCUs for all 1026 A5BS events (also for the 915 A3BS events separately) between each pair of adult samples. Correlations of ABS events in the same tissue between each pair of individuals were significantly higher than correlations in the same individual between each pair of tissues (Figure 2G). The abundance of circRNAs and their host genes also showed the same trend as ABS events: higher variation between different tissues of the same individual than between the same tissue from different individuals (Figure 2G). Furthermore, high fractions of tissue-specific A5BS and A3BS events were observed among most human adult tissues (Supplementary Figure S4C; the fractions were low for the tissues with low sequencing depths), further supporting that the differences among ABS events observed between tissue samples represent more cross-tissue variations than cross-individual variations.

### ABS events are more frequent and complex in brain tissues than in non-brain tissues

**Figure 3.**
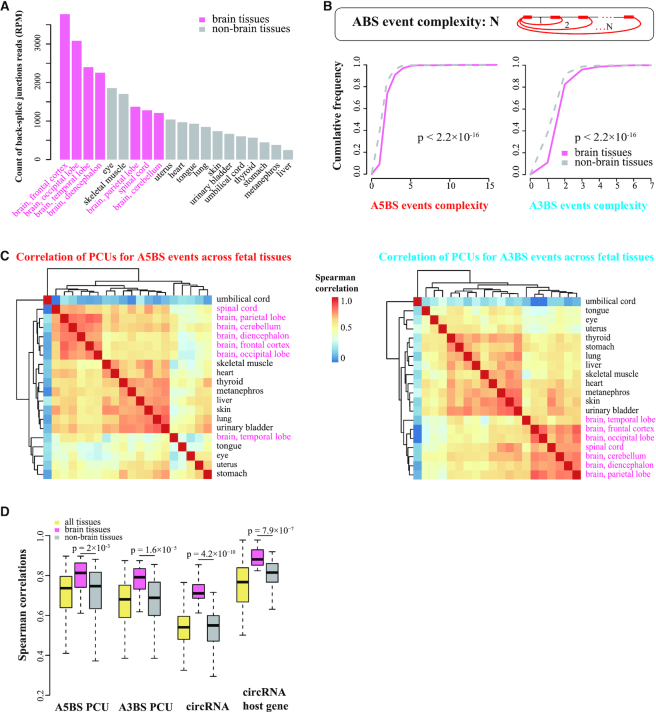
ABS events in human brain tissues and non-brain tissues. (**A**) The count of back-splice junction reads (in RPM) was shown for different fetal tissues, with a clear trend that brain tissues had more back-splice junction reads than non-brain tissues. (**B**) The top inset shows the definition of ABS event complexity. The cumulative frequency of A5BS events (left panel) and A3BS events (right panel) complexity in brain and non-brain tissues. (**C**) Average-linkage hierarchical clustering of human fetal tissues by Spearman correlation coefficient of PCUs for A5BS events (left panel) and A3BS events (right panel). (**D**) Spearman correlations of PCUs for ABS events, circRNAs, and their host genes among all 20 fetal tissues, 7 fetal brain tissues and 13 fetal non-brain tissues, respectively.

circRNAs are known to be highly enriched in brain tissues and neurons (27). Accordingly, we observed that among the 20 fetal tissues, the overall abundance of circRNAs was higher in brain tissues than in non-brain tissues (Figure 3A). (None of the 18 adult tissues was from the brain; thus, we focused our analysis in this section on the 20 fetal tissues.) We showed above that tissues with more circRNA reads had proportionally higher fractions of ABS circRNAs (Figure 1C; Supplementary Figure S1B), and the brain samples indeed contained the highest fractions of ABS circRNAs (Supplementary Figure S1C). Furthermore, the complexity of ABS events, defined as the number of different circRNAs in an ABS event, was higher in brain tissues than in non-brain tissues (Figure 3B; Wilcoxon signed-rank test *P*-values < 2.2 × 10^−16^ for both A5BS and A3BS events).

We then asked whether brain tissues exhibited specific ABS patterns. To answer this question, we computed Spearman correlation coefficients using the PCUs of ABS events between all pairs of fetal tissues, followed by hierarchical clustering. Brain tissues formed a distinct cluster for both A5BS and A3BS events, with the exception of the temporal lobe (Figure 3C). For comparison, we also performed hierarchical clustering on the abundance of circRNAs and their host genes and reached the same conclusion: brain tissues clustered together and segregated from non-brain tissues (Supplementary Figure S5A, B). Consistently, brain tissues had higher correlations than non-brain tissues for PCUs, circRNAs and circRNA host genes (Figure 3D).

### The abundance of *Alu* elements is positively correlated with ABS event complexity

**Figure 4.**
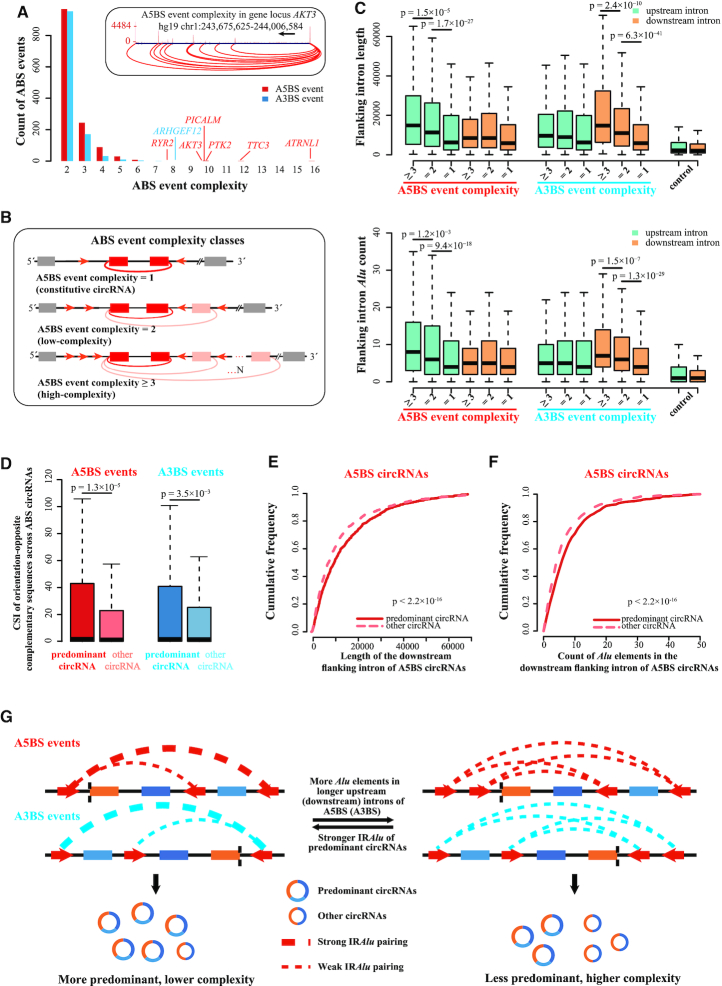
The count of *Alu* elements and flanking intron length positively correlated with ABS event complexity. (**A**) The distribution of ABS event complexity in human fetal tissues. The top inset shows an example A5BS event with high complexity produced from the *AKT3* gene in human occipital lobe tissue. (**B**) Schematic illustration of three ABS event complexity classes. Colored boxes, exons. Black lines, introns. Colored arcs, back-splicing. (**C**) (Top panel) the upstream and downstream flanking intron length of different ABS event complexity classes, respectively. (Bottom panel) the count of *Alu* elements in upstream and downstream flanking introns for different ABS event complexity classes, respectively. (**D**) The Complementary Sequence Index (CSI) of orientation-opposite complementary sequences across ABS predominant and other circRNAs separately. (**E**) The cumulative frequency of downstream flanking intron length of A5BS circRNAs. (**F**) The cumulative frequency of *Alu* element count in the downstream flanking intron of A5BS circRNAs. (**G**) A schematic diagram illustrates a model of how the competition of *Alu* element pairing regulates alternative back-splicing. Co-transcriptional back-splicing can also facilitate the competition between the predominant circRNA and other circRNAs via their sequence features. Colored boxes, exons. Black lines, introns. Colored arcs, back-splicing. Circles, circRNAs.

It is well known that reverse complementary sequences, especially *Alu* elements in circRNA-flanking introns, contribute greatly to circRNA formation (11,17). Furthermore, the competition among these *Alu* pairs was shown to modulate the formation of ABS events (12). We asked whether genomic features such as flanking intron length and *Alu* element count would have an effect on the complexity of ABS events (Materials and Methods). Most ABS events contain two circRNAs (complexity = 2), but some involve more than 10 circRNAs (Figure 4A). For example, an A5BS event in the human gene *AKT3* contained 10 high-confidence circRNAs (Figure 4A inset). To simplify the presentation of our analysis results, we divided ABS events into two classes by complexity, high (≥3) or low (=2). By this definition, constitutive circRNAs have the lowest complexity (=1) (Figure 4B). We found that, for A5BS events, the *upstream* introns flanking the high-complexity circRNAs were significantly longer than those flanking low-complexity circRNAs or constitutive circRNAs (Figure 4C, top). Accordingly, the *upstream* flanking introns of high-complexity, low-complexity, and constitutive circRNAs had the most, intermediate, and fewest numbers of *Alu* elements (Figure 4C, bottom). In sharp contrast, we did not observe significant differences in intron lengths or *Alu* counts among the *downstream* intron flanking these three complexity classes of A5BS events (Figure 4C). Conversely, when we examined A3BS events, we found that the *downstream* introns flanking the high-complexity circRNAs were significantly longer than those flanking low-complexity circRNAs or constitutive circRNAs (Figure 4C, top). This trend was consistent for *Alu* counts in these *downstream* introns of A3BS events (Figure 4C, bottom), but not for the intron length or *Alu* counts in the *upstream* flanking introns. Thus, the varying numbers of *Alu* elements in circRNA-flanking introns may contribute to the differential regulation of 5′ versus 3′ ABS events.

Furthermore, we explored three types of genomic features between the predominant circRNA and the other circRNAs in the same ABS event. First, we use Complementary Sequence Index (CSI) to quantify the RNA pairing capacity of reverse complementary sequences across introns that flank ABS circRNAs (Materials and Methods). We found that the CSI of the predominant circRNA was significantly higher than the other circRNAs in the same A5BS or A3BS events (Figure 4D). Second, considering that the circRNAs in an A5BS event share the same *upstream* flanking intron but have different downstream flanking introns, we compared the lengths of the *downstream* flanking introns for the predominant circRNA and the other circRNAs. We found that the intron of the predominant circRNA was significantly longer than the introns of other circRNAs (Figure 4E; Wilcoxon signed-rank test *P*-value < 2.2 × 10^−16^). As to circRNAs in an A3BS event, which share the same downstream flanking intron, we found that the upstream flanking intron for the predominant circRNA was longer than those for the other circRNAs (Supplementary Figure S5C; Wilcoxon signed-rank test *P*-value = 2.9 × 10^−3^). Third, the *downstream* flanking introns of A5BS predominant circRNAs contained significantly more *Alu* elements than other circRNAs in the corresponding A5BS events (Figure 4F; Wilcoxon signed-rank test *P*-value < 2.2 × 10^−16^) although this difference was not significant for the upstream flanking introns of A3BS circRNAs (Supplementary Figure S5D). We concluded that genomic features may dictate the choice of predominant circRNAs in ABS events.

## DISCUSSION

circRNAs are an abundant class of noncoding RNAs with a covalently closed loop structure primarily produced by back-splicing of protein-coding genes (28). They are widely detected in many tissues and their functions have been uncovered in recent years (1,6,29). Through alternative back-splicing, a locus can produce multiple circRNAs, sharing the same 3′ back-splice site (A5BS) or 5′ back-splice site (A3BS), with different abundance and complexity across cell types (12,30). An early study analyzed ABS events in a dozen cell lines (12). Here, we comprehensively analyzed ABS events in 37 human tissues. We found that ABS events were associated with most circRNAs and the ABS events tended to be tissue specific (Figures 1 and 2). We further showed that tissues from seven brain regions exhibited similar patterns of ABS events and the ABS events in brain tissues were more complex than those in non-brain tissues (Figure 3).

Although we experimentally tested the PCU values of two ABS events using RT-qPCR (Figure 2C and Supplementary Figure S3), more extensive experimental testing of additional ABS events, especially those ABS events that show high tissue specificity, would be desirable. While biosamples from the same type of tissue generally use the same circRNA as the predominant circRNA in ABS events (Figure 2B), the PCU values of the same ABS event can still vary between different donors (Figure 2; Supplementary Figure S4) and between fetal and adult tissues. Thus, validation efforts need to distinguish technical variations from biological variations, with the latter being different developmental stages or different individual donors.

The earlier ABS analysis in human cell lines (12) showed that ABS occurrence was correlated with the competitive pairing of *Alu* elements across flanking introns. Our results support this competition model and offer more mechanistic details on how the competition of *Alu* element pairing could regulate alternative back-splicing (Figure 4G). We found that longer *upstream* flanking introns were correlated with A5BS events with higher complexity while longer *downstream* flanking introns were correlated with A3BS events with higher complexity (Figure 4C). Based on these data, we propose that the longer introns could accommodate more *Alu* elements and intensify the competition of their pairing, leading to the corresponding A5BS and A3BS events with higher complexity. Further supporting our hypothesis, we observed that the predominant circRNAs had stronger *Alu* element pairs and longer introns, which could facilitate the corresponding *Alu* element pairing and help these circRNAs win the competition (Figure 4D-F; Supplementary Figure S5C, D).

Earlier studies (4,31) showed that a proportion of nascent circRNAs were detected only after their host genes was completely transcribed, i.e. post-transcriptionally. Because the biogenesis of circRNAs requires the canonical spliceosomal machinery (26) and splicing occurs cotranscriptionally for most genes, alternative back-splicing could occur co-transcriptionally for some circRNAs. Our results provide two lines of evidence that support the co-transcriptional model. First, we detected more A5BS events than A3BS events (Figure 1F; Supplementary Figure S2A, C, D), which supports cotranscriptional back-splicing because the 5′-to-3′ transcriptional direction would allow more time for the alternative selection of downstream 5′ back-splice sites than the alternative selection of upstream 3′ back-splice sites (Supplementary Figure S2E). Second, considering the competition model, co-transcriptional back-splicing can modulate the choice of back-splice sites during an ABS event. Intriguingly, the correlation between sequence features (intron length and *Alu* count) and complexity is more significant for A3BS events than for A5BS events (Figure 4C), suggesting that A3BS is more dependent on the sequence features than A5BS is, possibly because it is more difficult to accomplish A3BS than A5BS co-transcriptionally. Furthermore, we found that the CSIs of the predominant circRNAs were more significantly higher than the CSIs of other circRNAs for A5BS events than for A3BS events (*P* = 1.3 × 10^−5^ versus 3.5 × 10^−3^; Figure 4D). Similarly, the differences in length and *Alu* count of the downstream intron between the predominant A5BS circRNAs and the other A5BS circRNAs were highly significant (*P* < 2.2 × 10^−16^; Figure 4E, F) while they were much less or not significant for the upstream flanking introns of A3BS circRNAs (Supplementary Figure S5C, D). These data reinforce the model that co-transcriptional back-splicing facilitates the competition between the predominant A5BS circRNAs and other A5BS circRNAs via the sequence features in their downstream introns.

Despite having the same *cis*-elements, ABS events in different tissues exhibit tissue-specific patterns (Figures 2 and 3), suggesting the involvement of trans-factors in the regulation of alternative back-splicing. Previous studies revealed several RNA binding proteins (RBPs) involved in regulating the biogenesis of circRNAs. NF90/NF110 (two isoforms of the same protein) and DHX9 are two RBPs with a double-stranded RNA binding domain, and they can bind to the IR*Alu*s in the introns flanking circularized exons and regulate the production of circRNAs. NF90/NF110 was reported to stabilize IR*Alu*s and promote circRNA generation (32), while DHX9 destabilizes IR*Alu*s, leading to specific suppression of *Alu*-mediated circRNAs (33). Other RBPs like QKI (34), HNRNPL (35) and FUS (36) can bind to specific RNA motifs and regulate the abundance of circRNAs. The relatively long flanking introns of ABS circRNAs and their abundantly distributed *Alu* elements (Figure 4) offer more potential binding sites for RBPs to shape the tissue-specific alternative back-splicing patterns. The detailed mechanisms of action for these trans-factors still need to be explored.

In summary, we comprehensively identified ABS events in human tissue transcriptomes and provide a resource for the circRNA research community. Our results revealed the extent of ABS events in circRNAs and the genomic features associated with the regulation of ABS events, providing insights into the complexity of circRNA biogenesis, expression, and regulation.

## Supplementary Material

gkaa005_Supplemental_Files
