# Supplementary material for: Comprehensive identification of alternative back-splicing in human tissue transcriptomes
Source: Nucleic Acids Res. 2020 Jan 24;48(4):1779–89. doi: 10.1093/nar/gkaa005 (PMC7038940; doi:10.1093/nar/gkaa005)
Supplement: gkaa005_Supplemental_Files [file gkaa005_supplemental_files.zip › Supplementary_Figure.pdf]

Zhang et al., Supplementary Figure S1

A

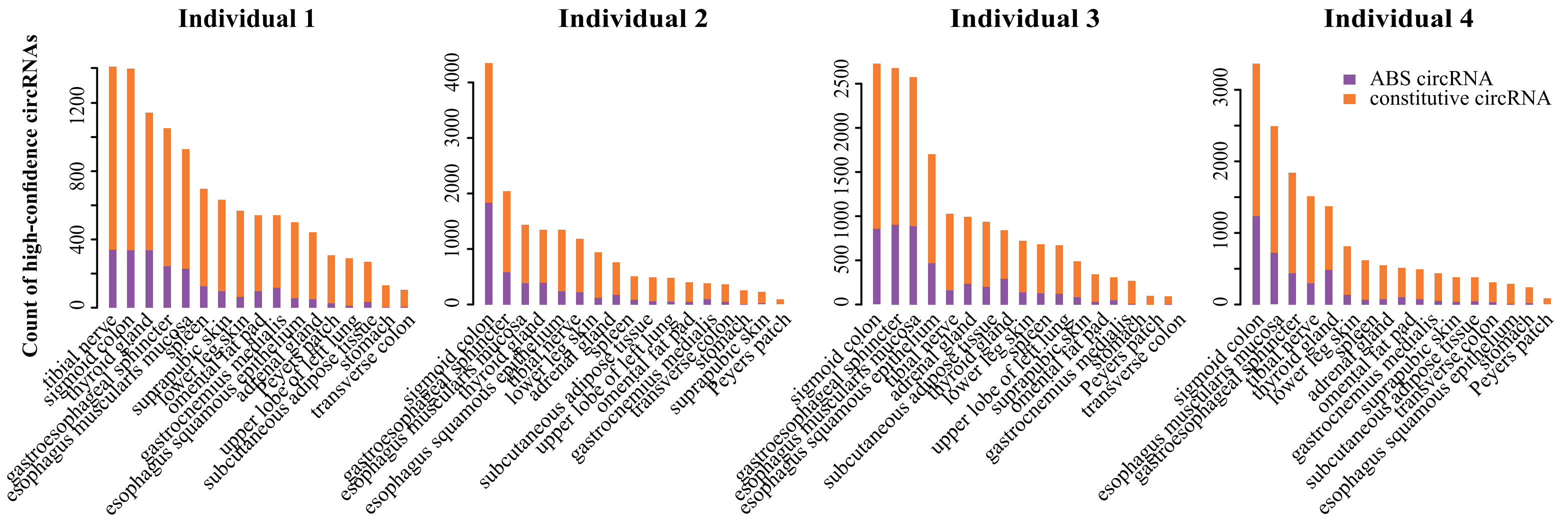

B

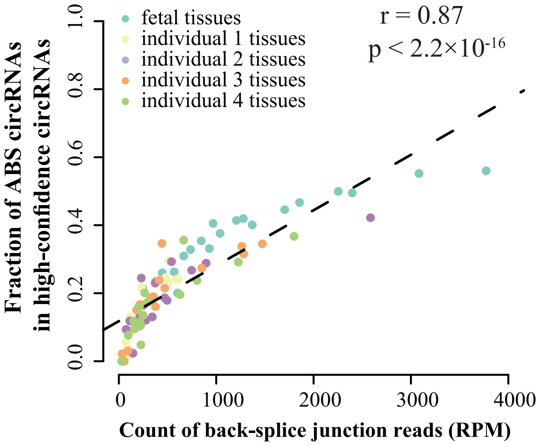

D

Binsize (circRNAs/bin)	A5BS or A3BS circRNA (ABS circRNA) fraction	A5BS circRNA fraction	A3BS circRNA fraction	A5BS & A3BS circRNA fraction
50	0.845	0.650	0.522	0.318
100	0.844	0.650	0.521	0.318
150	0.843	0.649	0.519	0.315
200	0.844	0.650	0.521	0.318
250	0.841	0.645	0.518	0.313
300	0.842	0.648	0.519	0.317
350	0.844	0.651	0.521	0.319
400	0.840	0.647	0.516	0.314
450	0.839	0.643	0.517	0.311
500	0.837	0.642	0.516	0.313
Mean	0.842	0.647	0.519	0.316
Std	0.003	0.003	0.002	0.003

C

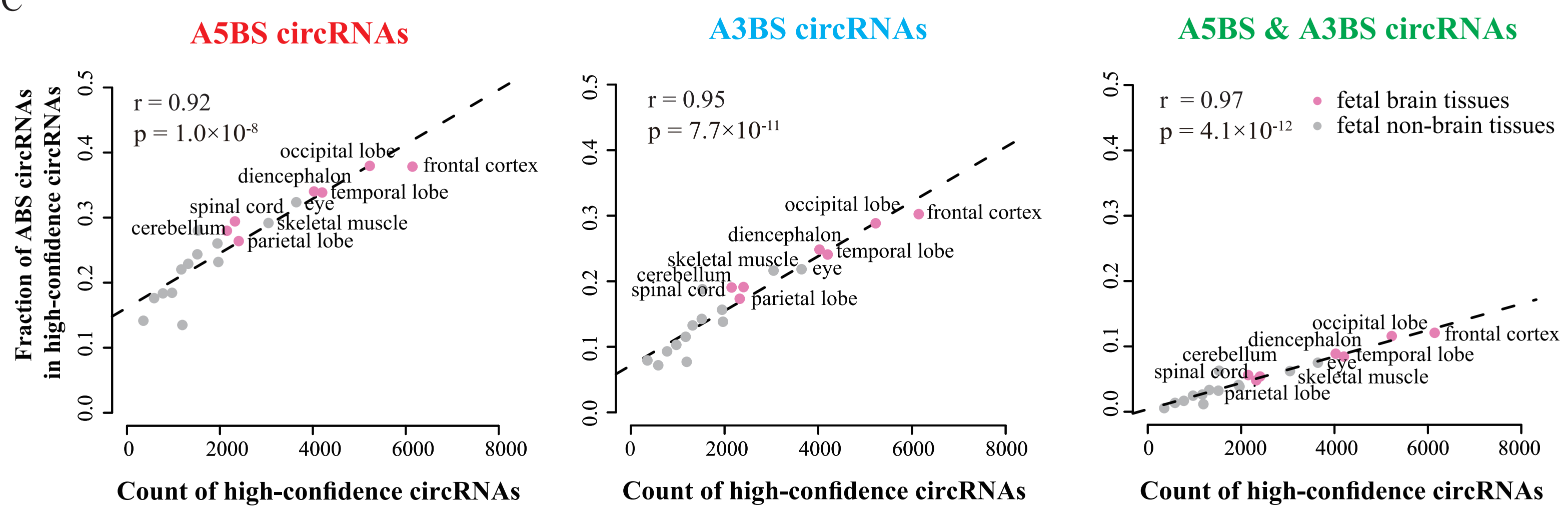

Supplementary Figure S1.

(A) The count of ABS and constitutive circRNAs among high-confidence circRNAs (RPM ≥ 0.1) for human adult tissues. (B) The fraction of ABS circRNAs in high-confidence circRNAs positively correlate with the count of normalized back-splice junction reads (in RPM) across human tissues (the r value was calculated by Pearson correlation). (C) The fraction of A5BS circRNAs, A3BS circRNAs, and A5BS & A3BS circRNAs in high-confidence circRNAs all positively correlated with the count of high-confidence circRNAs (r values were calculated by Pearson correlation). (D) Assess the effect of bin size on the estimation of the fraction of ABS circRNAs. The bin size was varied from 50 to 500 circRNAs per bin and the curve fitting was repeated. The mean and standard deviation of the resulting asymptotic ABS circRNAs fractions across the bin sizes are provided.

Zhang et al., Supplementary Figure S2

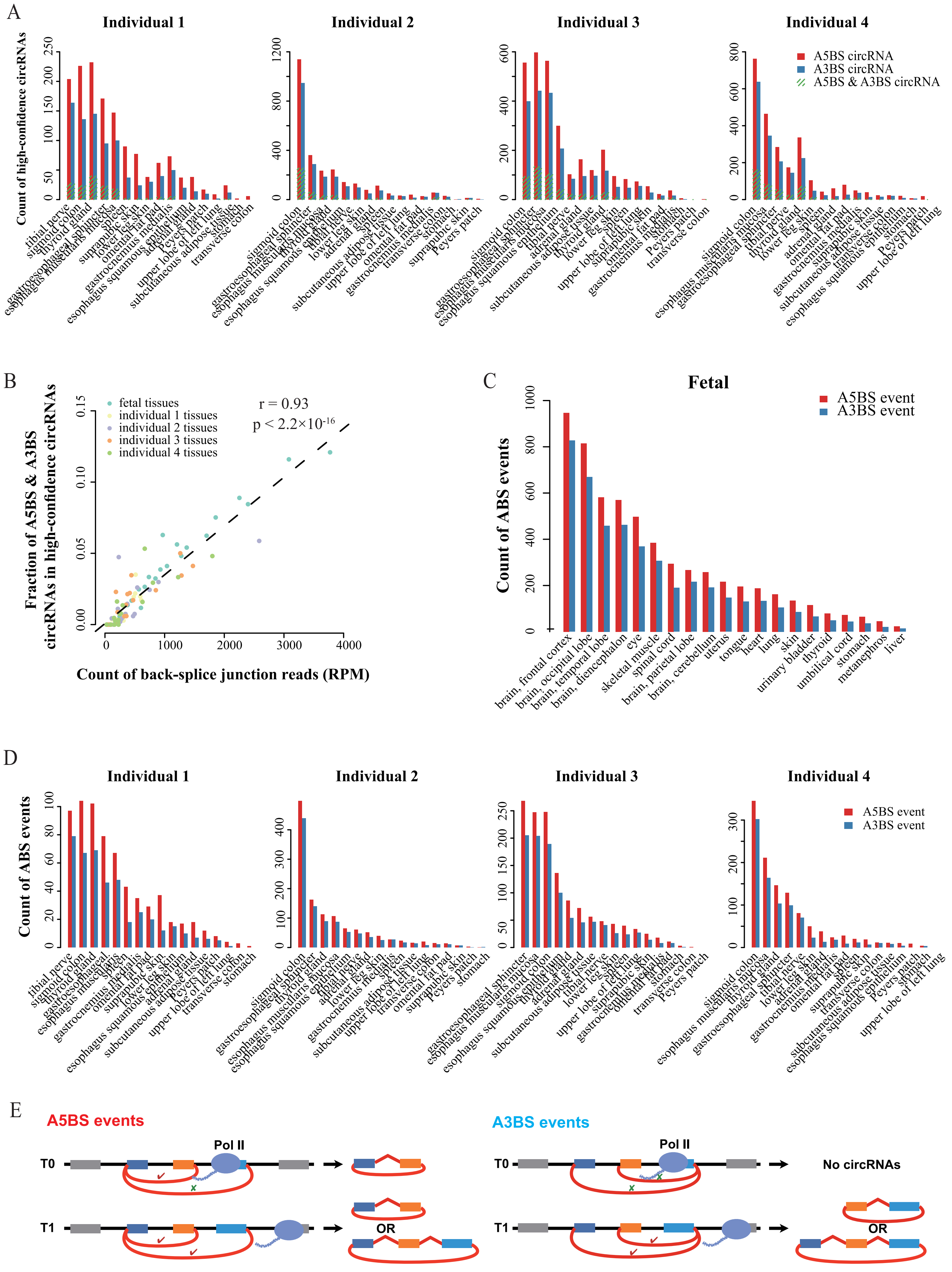

Supplementary Figure S2.

(A) The count of A5BS & A3BS circRNAs among high-confidence circRNAs (RPM $\geq$ 0.1) for human adult tissues. (B) The fraction of A5BS & A3BS circRNAs in high-confidence circRNAs positively correlate with the count of normalized back-splice junction reads (in RPM) across human tissues (the r value was calculated by Pearson correlation). (C) The count of high-confidence A5BS and A3BS events for human fetal tissues. (D) The count of high-confidence A5BS and A3BS events for human adult tissues. (E) A schematic diagram illustrates how the 5′-to-3′ transcriptional direction allows more time for the selection of alternative downstream 5′ back-splice sites than the selection of upstream alternative 3′ back-splice sites.

Zhang et al., Supplementary Figure S3

A

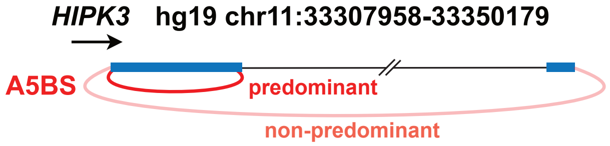

Uterus	predominant	non-predominant	PCU
adult 51 years	1338	31	97.7
adult 53 years	1371	15	98.9

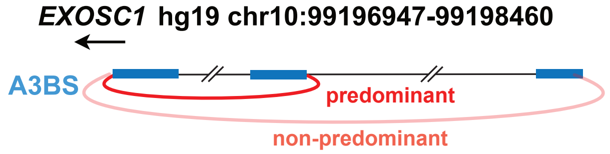

Uterus	predominant	non-predominant	PCU
adult 51 years	84	36	64.1
adult 53 years	220	66	76.9

B

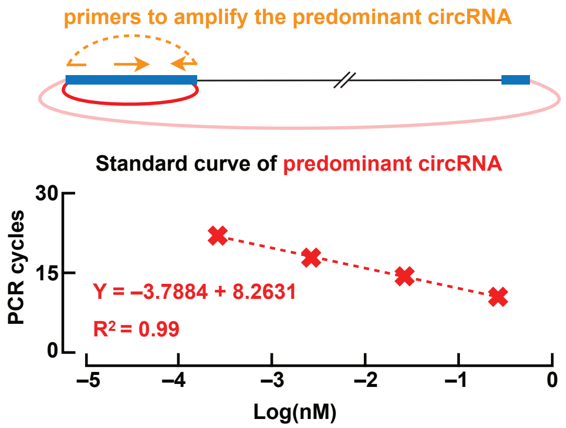

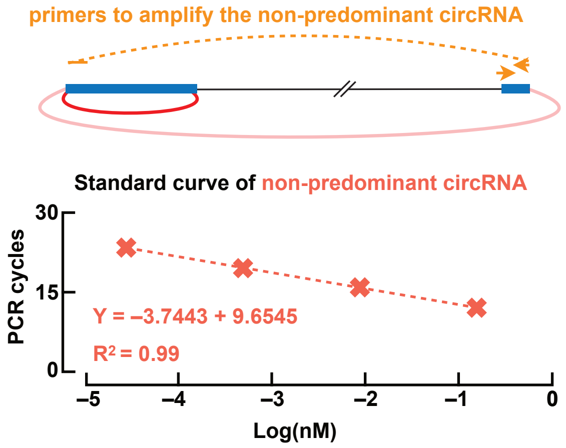

Uterus (adult)		predominant	non-predominant
Replicate 1	PCR cycles	22.10	27.34
	Copy number (nM)	2.223×10^{-4}	1.889×10^{-5}
Replicate 2	PCR cycles	22.09	27.42
	Copy number (nM)	2.245×10^{-4}	1.802×10^{-5}
Average	Copy number (nM)	2.234×10^{-4}	1.846×10^{-5}

Predominant circRNA% = 92.37%

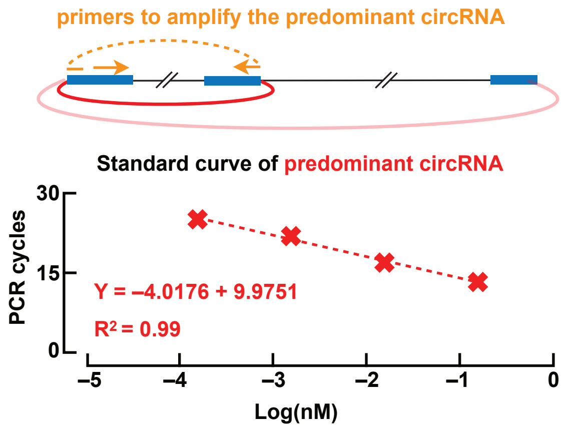

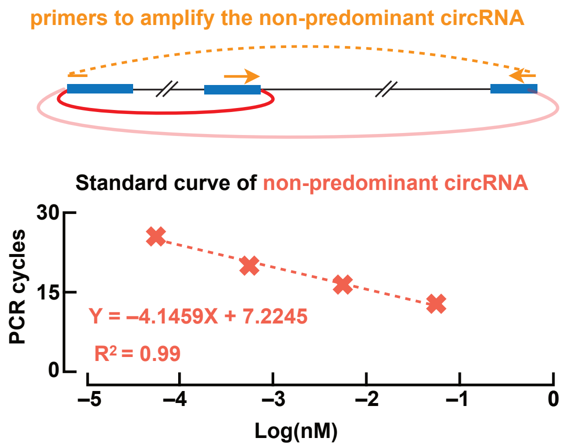

Uterus (adult)		predominant	non-predominant
Replicate 1	PCR cycles	30.30	30.70
	Copy number (nM)	8.715×10^{-6}	2.171×10^{-6}
Replicate 2	PCR cycles	30.52	30.48
	Copy number (nM)	7.703×10^{-6}	2.454×10^{-6}
Average	Copy number (nM)	8.209×10^{-6}	2.313×10^{-6}

Predominant circRNA% = 78.02%

Supplementary Figure S3.

(A) The PCUs of an A5BS event in the *HIPK3* gene (left panel) and an A3BS event in the *EXOSC1* gene (right panel) were computed based on the RNA-seq data in the uterus tissue from two adult human donors. (B) Validation of the PCUs in (A) using quantitative real-time PCR with primers specifically designed for each circRNA. A real-time PCR standard curve is shown for each circRNA, the copy numbers of the predominant circRNA and the non-predominant circRNA are calculated based on the standard curves, and the resulting PCU is determined accordingly.

Zhang et al., Supplementary Figure S4

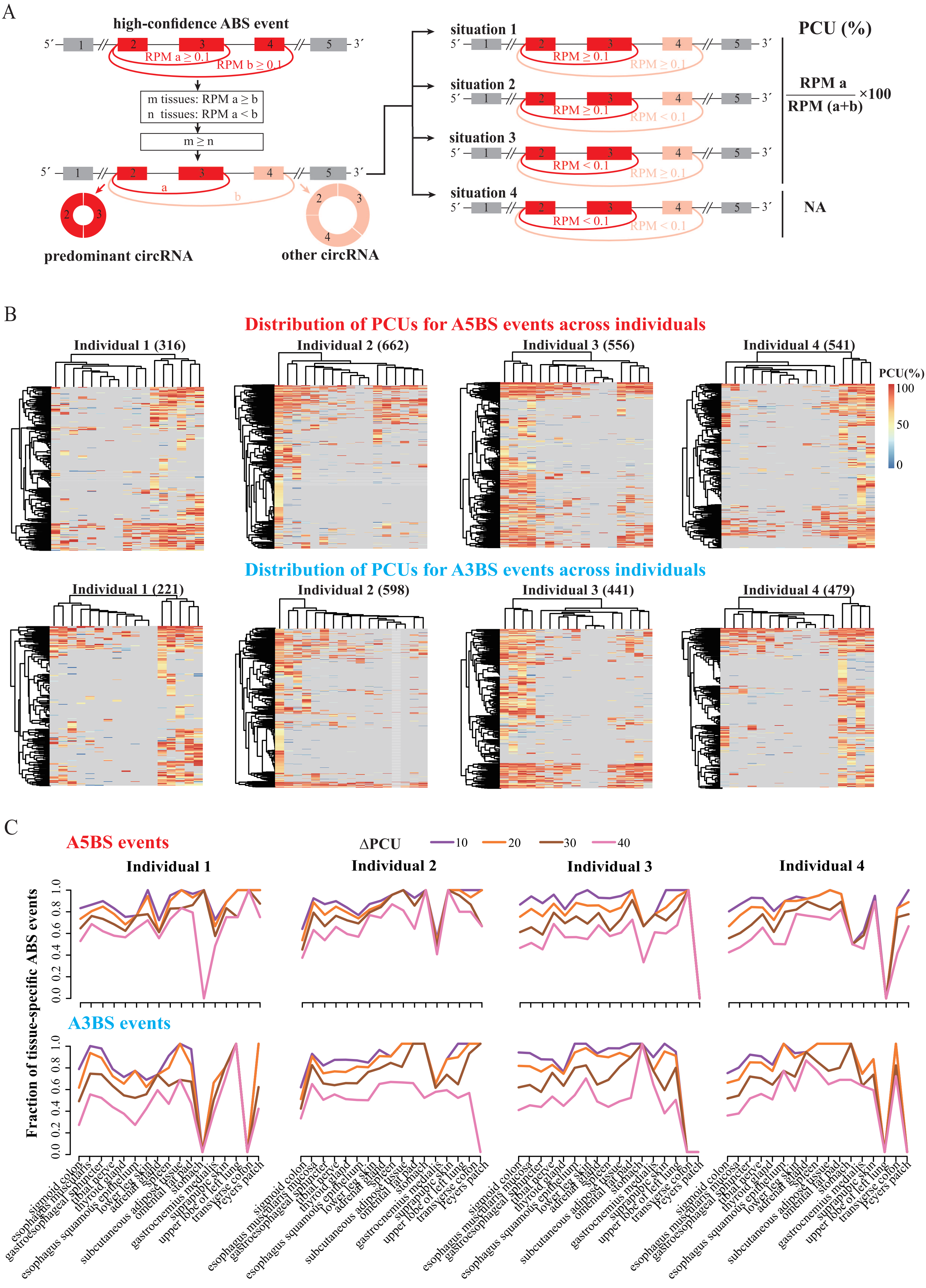

Supplementary Figure S4.

(A) A schematic diagram explaining the definition of predominant and other circRNAs for high-confidence ABS events, which contain two or more highly expressed circRNAs (≥ 0.1 RPM) in at least one tissue (left panel). The four situations when calculating PCU for high-confidence ABS events in human tissues are also shown (right panel). Colored boxes, exons. Black lines, introns. Colored arcs, back-splicing. Circles, circRNAs. (B) The distribution of PCUs for A5BS events and A3BS events across human adult tissues. (C) The fraction of tissue-specific A5BS events and A3BS events among each human adult tissue with ΔPCU ranging from 10 to 40.

Zhang et al., Supplementary Figure S5

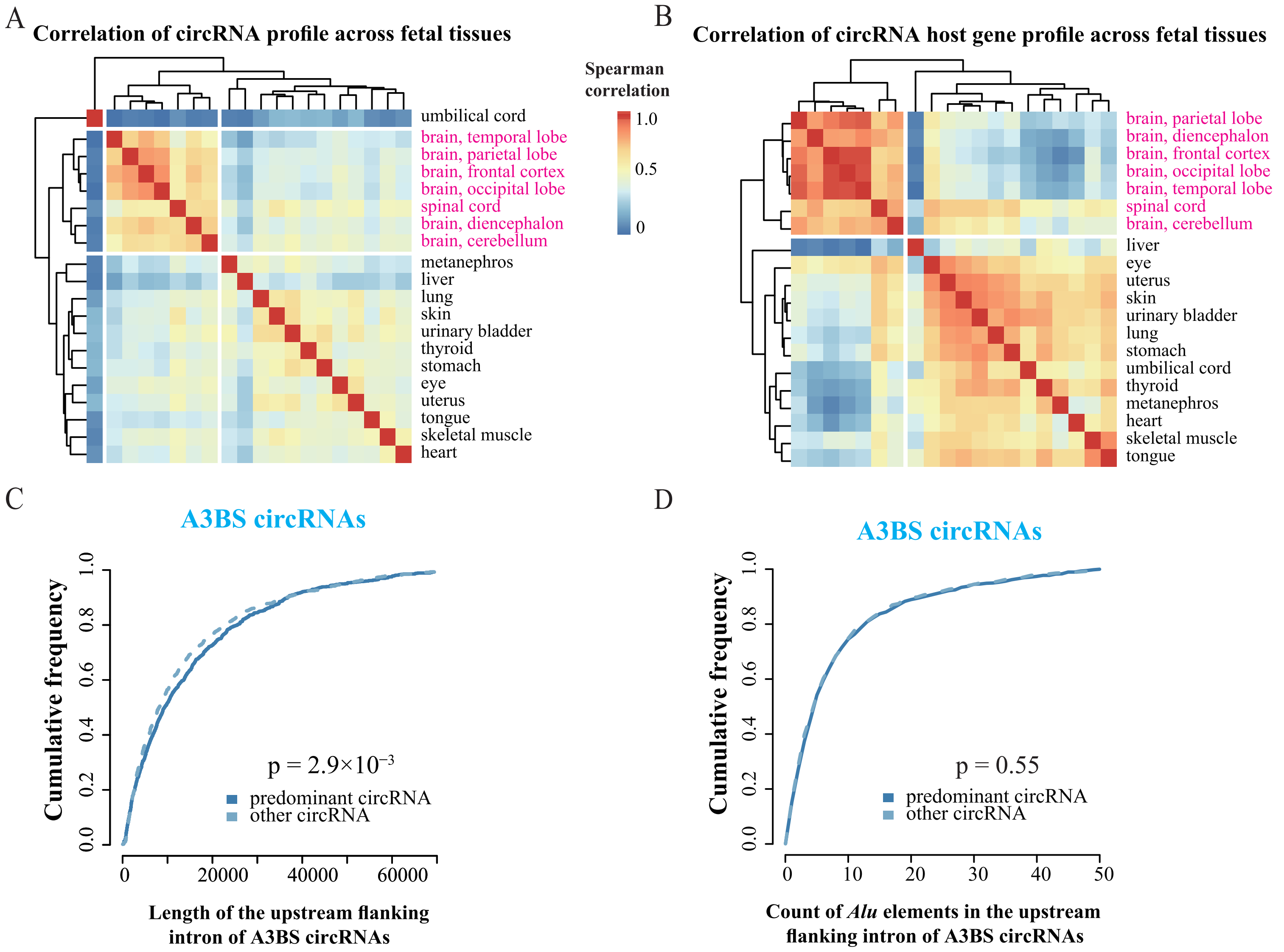

Supplementary Figure S5.

(A; B) Average-linkage hierarchical clustering of human fetal tissues by Spearman correlation coefficient of circRNA profile (A) and circRNA host gene profile (B). (C) The cumulative frequency of the upstream flanking intron length of A3BS circRNAs. (D) The cumulative frequency of *Alu* element count in the upstream flanking introns of A3BS circRNAs.
