# Supplementary material for: Comprehensive identification of alternative back-splicing in human tissue transcriptomes
Source: Nucleic Acids Res. 2020 Jan 24;48(4):1779–89. doi: 10.1093/nar/gkaa005 (PMC7038940; doi:10.1093/nar/gkaa005)
Supplement: gkaa005_Supplemental_Files [file gkaa005_supplemental_files.zip › Supplementary_Table_S4.docx]

**Supplementary Table S4**.  Primer sequences used to validate alternative back-splicing events.

(A) Primer sequences used to amplify specific circRNAs of the A5BS event in *HIPK3* gene

HIPK3-predominant-circRNA-F: CATGCTGATCTCAAGCCAGA

HIPK3-predominant-circRNA-R: GAGGCCATACCTGTAGTACCGAGA

HIPK3-non-predominant-circRNA-F: TATTGGGGTTGCCATTTTGT

HIPK3-non-predominant-circRNA-R: GAGGCCATACCTGATCATACTCC

(B) Primer sequences used to amplify specific circRNAs of the A3BS event in *EXOSC1* gene

EXOSC1-predominant-circRNA-F: CCACAGTGAGTCAGGTTGAAATTTAT

EXOSC1-predominant-circRNA-R: GCGGTGGTTAGCAGGTAGTT

EXOSC1-non-predominant-circRNA-F: ACAGTGAGTCAGCAAGGAAGATG

EXOSC1-non-predominant-circRNA-R: TTTGGCCAAGACAATGTCAC
